# The Photodynamic Anticancer and Antibacterial Activity Properties of a Series of *meso*-Tetraarylchlorin Dyes and Their Sn(IV) Complexes

**DOI:** 10.3390/molecules28104030

**Published:** 2023-05-11

**Authors:** Rodah Soy, Balaji Babu, John Mack, Tebello Nyokong

**Affiliations:** 1 Institute for Nanotechnology Innovation, Rhodes University, Makhanda 6140, South Africa; 2 Department of Chemistry, SRM University-AP, Amaravati 522502, India

**Keywords:** chlorins, Sn(IV) complexes, photodynamic therapy, photodynamic antimicrobial chemotherapy, photosensitizer dyes, singlet oxygen

## Abstract

A series of tetraarylchlorins with 3-methoxy-, 4-hydroxy- and 3-methoxy-4-hydroxyphenyl *meso*-aryl rings (**1**-**3**-Chl) and their Sn(IV) complexes (**1**-**3**-SnChl) were synthesized and characterized so that their potential utility as photosensitizer dyes for use in photodynamic therapy (PDT) and photodynamic antimicrobial chemotherapy (PACT) can be assessed. The photophysicochemical properties of the dyes were assessed prior to in vitro PDT activity studies against MCF-7 breast cancer cells through irradiation with Thorlabs 625 or 660 nm LED for 20 min (240 or 280 mW·cm^−2^). PACT activity studies were performed against both planktonic bacteria and biofilms of Gram-(+) *S. aureus* and Gram-(−) *E. coli* upon irradiation with Thorlabs 625 and 660 nm LEDs for 75 min. The heavy atom effect of the Sn(IV) ion results in relatively high singlet oxygen quantum yield values of 0.69−0.71 for **1**-**3**-SnChl. Relatively low IC_50_ values between 1.1−4.1 and 3.8−9.4 µM were obtained for the **1**-**3**-SnChl series with the Thorlabs 660 and 625 nm LEDs, respectively, during the PDT activity studies. **1**-**3**-SnChl were also found to exhibit significant PACT activity against planktonic *S. aureus* and *E. coli* with Log_10_ reduction values of 7.65 and >3.0, respectively. The results demonstrate that the Sn(IV) complexes of tetraarylchlorins merit further in depth study as photosensitizers in biomedical applications.

## 1. Introduction

Cancer causes more deaths each year than malaria, tuberculosis, and HIV combined [1]. Traditional treatments can have severe side effects. In the mid-1970s, photodynamic therapy (PDT) emerged as an alternative non-invasive treatment option for treating cancers that can be readily reached by laser light via a fiber optic. Upon photoexcitation, the T_1_ triplet state of a photosensitizer (PS) dye is populated through intersystem crossing and singlet oxygen is generated as the main cytotoxic agent from molecular dioxygen by energy transfer [2]. Photofrin^®^ was the first PS dye to be clinically approved. It is comprised of a series of porphyrin oligomers that exhibit significant dark toxicity and slow clearance from the body. The lowest energy Q band is relatively weak and only absorbs at the edge of the therapeutic window (620–850 nm), where human tissue has high transparency to incident laser light. Endogenous chromophores, such as heme, absorb significantly at <600 nm [3], while H_2_O absorbs significantly at longer wavelengths in the near-infrared (NIR) region. Other optical properties, such as the scattering coefficients of particles in biological tissues, exhibit a strong wavelength dependence at higher energy. As a result, new PS dyes for PDT should absorb significantly in the phototherapeutic window.

**Scheme 1:**
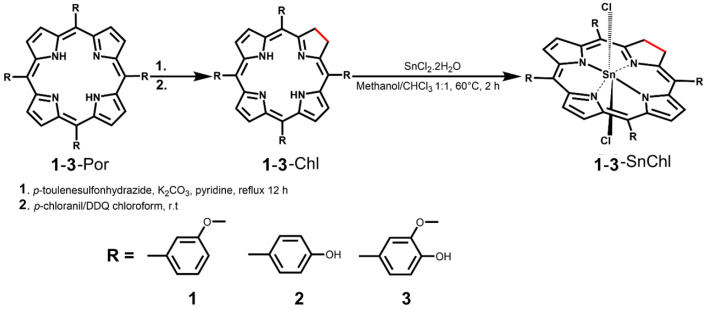
Synthesis of the chlorin dyes (**1**-**3**-Chl) and their Sn(IV) complexes (**1**-**3**-SnChl) from the corresponding porphyrins (**1**-**3**-Por). The red bond highlights the reduced peripheral pyrrole bond of the chlorins.

The second generation of PS dyes was mainly comprised of phthalocyanines, since their intense Q bands lie in the 650−700 nm range [4]. These dyes have solubility issues related to π-π stacking related aggregation, which can make their photophysicochemical properties less suitable for PS dye applications. Attempts have been made to address this by modifying the structures of PS dyes to enhance the singlet oxygen quantum yield (Φ_Δ_) and decrease aggregation effects. Chlorins (Scheme 1), such as chlorin e6 and Purlytin^®^ [5,6,7], have emerged as PS dyes for PDT due to their relatively intense Q bands at ca. 650 nm [3,8,9]. In recent years, we have reported a series of studies on the PDT activity properties of the Sn(IV) complexes of readily synthesized porphyrin analogues [10], including N-confused porphyrins [11,12], corroles [13,14], and chlorins [15,16,17]. This research has focused on Sn(IV) complexes, since the presence of two axial ligands through *trans*-axial on the Sn(IV) ion hinders π-π stacking and hence aggregation, and the heavy atom effect of this ion enhances the generation of singlet oxygen [10].

Our previous studies on chlorins have focused on ligands with *meso*-thienyl and –methylthiophenyl substituents [15,16,17]. In this study, the PDT activities of the Sn(IV) complexes of a series of structurally analogous chlorins with 3-methoxy-, 4-hydroxy- and 3-methoxy-4-hydroxyphenyl *meso*-aryl rings (**1**-**3**-SnChl) are compared to the corresponding chlorins (**1**-**3**-Chl), which were prepared by reducing one of the peripheral pyrrole bonds of the corresponding porphyrins (**1**-**3**-Por) (Scheme 1). The suitability of **1**-**3**-SnChl for use in photodynamic antimicrobial chemotherapy (PACT) [18,19,20] is also examined against both planktonic *S. aureus* and *E. coli* and their biofilms as typical Gram-(+) and Gram-(−) strains, respectively. Unusually for non-cationic species [21,22], high Log_10_ reduction values were reported against *E. coli* in our previous studies involving Sn(IV) chlorins with *meso*-aryl substituents that contain sulfur atoms [16,17].

## 2. Results

### 2.1. Synthesis and Characterization

**1**-**3**-Por and **2**-Chl have been reported previously, and were prepared according to literature procedures [23,24]. The target **1**-Chl and **3**-Chl free-base chlorins were synthesized from **1**-Por and **3**-Por by reducing one of their peripheral pyrrole bonds via the Whitlock diimide reduction method [25] (Scheme 1). **1**-**3**-Chl were metalated using SnCl_2_·2H_2_O to afford the target **1**-**3**-SnChl complexes at relatively high yield (Scheme 1).

The ^1^H NMR and MALDI-TOF MS data confirmed the structures of target chlorin dyes **1**-Chl and **2**-Chl and Sn(IV) complexes **1**-**3**-SnChl (Figures S1 and S2). The anticipated proton signals for the synthesized chlorin dyes can be readily assigned. The chemical shifts at 8.00−8.71 ppm in each ^1^H NMR spectrum are assigned to the six protons from the β-pyrrole positions, while the aromatic protons from the *meso*-aryl groups lie between 7.00−8.00 ppm at slightly lower frequencies. The hydroxy (OH) protons from the *meso*-aryl substituent groups lie at higher frequencies between 8.84−10.00 ppm for **2**-**3**-Chl and the **2**-**3**-SnChl complexes (Figure S1). The methyl (CH_3_) protons from the *meso*-aryl substituent groups lie between 3.83−4.07 ppm in the aliphatic region for **1**-Chl and **3**-Chl and their corresponding Sn(IV) chlorin complexes (Figure S1). The characteristic four pyrroline protons for chlorin dyes lie between 4.12−4.37 ppm as singlet signals (Figure S1). These proton peaks confirm the synthesis of chlorin dyes from their parent porphyrin structures [26,27]. Singlet peaks for the two NH core protons for **1**-**3**-Chl lie between −1.42 to −1.57 ppm in the negative low-frequency region. Upon insertion of Sn(IV) ions, the two NH protons disappeared in the spectra of **1**-**3**-SnChl (Figure S1). This confirms their successful synthesis. MALDI-TOF mass data confirmed the structures for the target chlorin dyes. The anticipated parent ion peaks are observed, which agree closely with the theoretical masses of chlorin dyes. The MALDI-TOF data for **1**-**3**-Chl and their **1**-**3**-SnChl complexes are provided in Figure S2. The MS data for **1**-**3**-Chl free-base chlorin dyes exhibit mainly [M+H]^+^ or [M]^+^ molecular ion species, while the **1**-**3**-SnChl complexes exhibited [M−Cl+H]^+^ or [M−Cl]^+^ parent peak species due to the loss of a chloride ion.

### 2.2. Photophyiscochemical Properties

**Figure 1:**
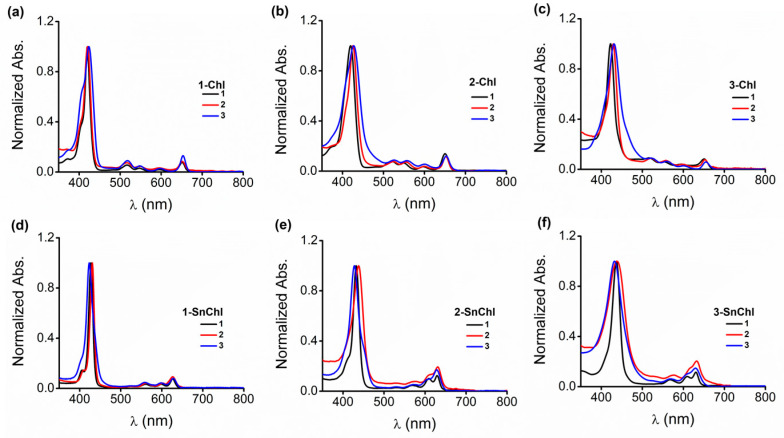
UV-visible absorption spectra of (**a**) **1**-Chl, (**b**) **2**-Chl, (**c**) **3**-Chl, (**d**) **1**-SnChl, (**e**) **2**-SnChl and (**f**) **3**-SnChl in CHCl_3_ (**1** black), DMSO (**2** red), 1% DMSO-water (**3** blue). Solutions were prepared at ca. 10 µM.

The ground state absorption spectra of the studied A_4_ free base *meso*-tetraaryl chlorins are typical of chlorin dyes, with intense B bands at ca. 425 nm and a set of four Q bands at low energy region and a characteristic slightly more intense Q band at ca. 650 nm, in contrast to the parent porphyrin dyes [28] (Figure 1). For Sn(IV) chlorins (**1**-**3**-SnChl), there are red shifts in the B bands, and the four Q bands of free-base ligands collapse into three with the characteristic slightly more intense Q band blue shifting to ca. 630 nm (Figure 1). The greater intensity of the Q band arises from hydrogen reduction on one of the peripheral β-β pyrrole double bonds of the parent porphyrin ring to form a low-symmetry chlorin dye [16,28]. Table 1 summarizes the optical properties of the chlorin dyes studied in DMSO. There are minor spectral changes due to inductive and mesomeric effects from the *meso*-aryl substituents of the chlorins studied. The absorption spectra of *meso*-4-hydroxyphenyl (**2**) and *meso*-4-hydroxy-3-methoxyphenyl (**3**) chlorin derivatives are red shifted in contrast to those of the *meta*-substituted *meso*-3-methoxyphenylchlorin (**1**). This is due to more favorable mesomeric interactions between the chlorin core and *meso*-phenyl groups with *para*-substituents [29].

The solvatochromic absorption data in CHCl_3_ and 1% DMSO/H_2_O of the chlorins studied are provided in Figure 1. The main absorption bands of the chlorin dyes studied exhibit minor solvatochromic effects with a slight dependence on solvent polarity, as evidenced by slight red shifts from CHCl_3_ < DMSO. Band broadening is observed in the absorption spectra of free-base chlorins **1**-**2**-Chl in 1% DMSO/H_2_O relative to those of the **1**-**2**-SnChl dichlorotin(IV) chlorin complexes (Figure 1 and Table S1), but is relatively limited for both sets of dyes. This is likely related to aggregation effects, which are decreased by the dichloro axial ligands of Sn(IV) chlorin complexes since face-to-face π-π stacking is hindered [10]. The additional broadening that is observed in the spectra of **3**-Chl and **3**-SnChl may be related to the hydrogen bonding of the 4-hydroxy-3-methoxyphenyl rings.

**Figure 2:**
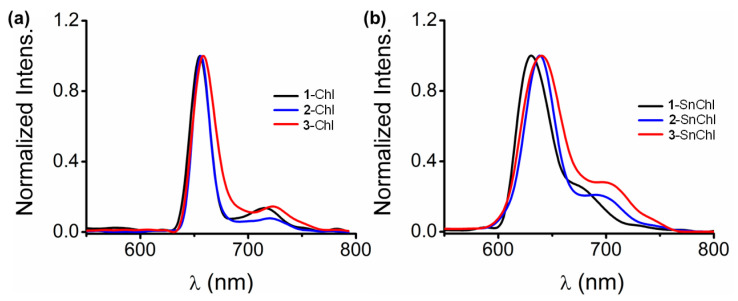
Fluorescence spectra of (**a**) **1**-**3**-Chl and (**b**) **1**-**3**-SnChl in DMSO. Solutions were prepared at ca. 0.2 µM and were excited at the B band maxima.

The chlorins studied have two-band emission profiles typical of chlorin dyes (Figure 2) [28]. The emission spectra of Sn(IV) chlorins are blue shifted in contrast to the free-base chlorins due to the introduction of the Sn(IV) ion resulting in rigid planar structures. The trends in the emission profiles of the chlorin dyes studied correspond closely to those in the UV-visible absorption spectra, with red shifts observed for **2**-Chl, and **3**-Chl relative to **1**-Chl (Figure 1, Table 1).

The photophysicochemical properties of **1**-**3**-Chl and **1**-**3**-SnChl in DMSO are summarized in Table 2. The free-base chlorins (**1**-**3**-Chl) have relatively low fluorescence quantum yield (Φ_F_) values and relatively long fluorescence lifetimes (τ_F_) (Table 2). This is consistent with the reported trends for chlorin dyes and is related to their flexible structures [30,31,32]. The Φ_F_ and τ_F_ values decreased significantly on metalation of the dyes to form **1**-**3**-SnChl due to the Sn(IV) ion heavy atom effect [16]. The triplet lifetime (τ_T_) values of **1**-**3**-Chl are also longer than those of **1**-**3**-SnChl due to the heavy atom effect [16]. The free-base chlorins (**1**-**3**-Chl) have relatively high Φ_Δ_ values that lie in the 0.59−0.62 range (Table 2). This can be attributed to effective quenching processes in the transfer of energy by the triplet state of chlorin dyes to molecular oxygen [31]. The Φ_Δ_ values of **1**-**3**-SnChl, on the other hand, are higher than those of the corresponding free-base chlorins due to the heavy atom effect [16]. The photostabilities in 1% DMSO/H_2_O solutions of the chlorins studied were determined by determining the extent of photobleaching of the B band after 30 min photoirradiation with a 660 nm (280 mW·cm^−2^) Thorlabs LED lamp mounted onto a Modulight 7710-680 medical laser system housing in a similar manner to the PDT and PACT activity measurements (Table 2). The free-base chlorins have low photostability values (63–67%), in contrast to those of their Sn(IV) chlorin complexes. This is due to the flexible structural properties of free-base chlorins since they lack the stabilization effect at the frontier π-MOs from a high valent metal ion [28]. The photostabilities of Sn(IV) chlorins are higher due to their greater structural rigidity and the heavy atom effect [16].

### 2.3. PDT Activities

**Figure 3:**
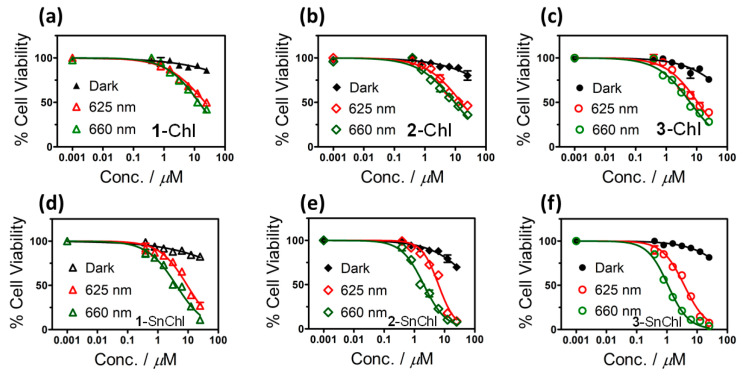
Cytotoxicity plots of MCF-7 cancer cells in the dark (black line) and under illumination for 20 min centered at 625 nm (red line) and 660 nm (green) with M625L3 and M660L4 Thorlabs LEDs, respectively, upon treatment with gradient concentrations of (**a**) **1**-Chl, (**b**) **2**-Chl, (**c**) **3**-Chl, (**d**) **1**-SnChl, (**e**) **2**-SnChl and (**f**) **3**-SnChl. The error bars represent the mean ± standard deviation of three independent assays each with three replicates.

Figure 3 provides the cytotoxicity plots of MCF-7 cancer cells in the dark and under 20 min illumination from Thorlabs M625L3 and M660L4 LEDs upon treatment with gradient concentrations of **1**-**3**-Chl and **1**-**3**-SnChl. Table 3 provides a summary of IC_50_, phototoxicity index (PI), and cell viability values of **1**-**3**-Chl and **1**-**3**-SnChl in the dark and under illumination. **1**-**3**-Chl and **1**-**3**-SnChl have IC_50_ values > 25 µM in the dark and ≥70% viable cells at 25 µM of the dyes. This suggests that **1**-**3**-Chl and **1**-**3**-SnChl are relatively innocuous toward MCF-7 cancer cells in the absence of illumination. This suggests that the chlorin dyes studied are promising candidates for use as PS dyes in PDT. During the PDT studies, fixed light doses of 280 and 336 J·cm^2^ were used to illuminate chlorin dyes with the 625 and 660 nm LEDs, respectively. The PDT results demonstrate that illumination of **1**-**3**-Chl and **1**-**3**-SnChl centered at 660 nm resulted in enhanced PDT activities relative to illumination centered at 625 nm (Table 3). This could be due to the reported favorable effects of stronger absorption of light by a PS dye at longer wavelength deep into the therapeutic window, since it can readily penetrate into the tissues resulting in enhanced phototherapy effects [16,33,34].

**1**-**3**-Chl free-base chlorins also exhibited relatively favorable PDT activities (Table 3). For example, **1**-**3**-Chl have relatively IC_50_ values of 10.8−22.4 µM, phototoxicity index (PI) values of 1.5−2.3, and cell viability values of 38.7−49.8% at 25 µM after illumination at 625 nm with significantly lower IC_50_ values of 6.1−13.9 µM after illumination at 660 nm (Table 3). As would be anticipated based on their high Φ_Δ_ values and stronger absorption in the NIR region, **1**-**3**-SnChl significantly outperformed their **1**-**3**-Chl free-base chlorin ligands (Table 3). For instance, upon illumination at 660 nm (Figure 3, Table 3), **1**-**3**-SnChl had IC_50_ values of 1.1−4.1 µM, and PI values of 16.7−24.8, and low cell viability values of 3.9−11.1% at 25 µM. The PDT activity trends due to the different *meso*-aryl substituents of **1**-**3**-Chl and **1**-**3**-SnChl are consistent with the anticancer properties of vanillic and phenolic derivatives [35,36,37]. For example, *meso*-vanillic chlorin derivatives (**3**-Chl, **3**-SnChl) followed by phenolic chlorins (**2**-Chl, **2**-SnChl) have significantly higher PDT activities than the *meso*-methoxyphenylchlorins (**1**-Chl and **1**-SnChl). 

### 2.4. Lipophilicity

The lipophilicity (Log P_o/w_) values of **1**-**3**-SnChl were determined by the shake flask method (see Section 4.6), since relatively low IC_50_ values were obtained (Table 3). The Log P_o/w_ values lie between 0.96 and 1.39 (Table 4) in the optimal lipophilic balance range for drugs [38], since the dichloro axial ligands can minimize aggregation effects at higher concentrations of the dyes and enhance cellular uptake [10,39,40]. The lower Log P_o/w_ values for **2**-SnChl and **3**-SnChl can be explained by the scope for hydrogen bonding with water through the -OH groups on the *meso*-aryl rings.

### 2.5. In Vitro PACT Cytotoxicity Studies against the Planktonic Cells of S. aureus and E. coli Bacteria

**Figure 4:**
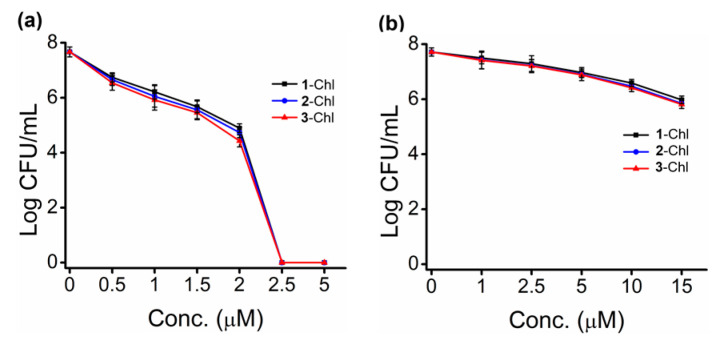
The cell viability plots for planktonic cells of (**a**) *S. aureus* and (**b**) *E. coli* upon treatment with gradient concentrations of **1**-**3**-Chl after 45 min illumination with a Thorlabs M625L3 (240 mW·cm^−2^) LED. The error bars represent the mean ± standard deviation of three independent assays each with three replicates.

Optimal concentrations of 1 µM for *S. aureus* and 5 µM for *E. coli* were selected for use during time-dependent studies for **1**-**3**-Chl and **1**-**3**-SnChl based on concentration studies (Figure 4). Table 5 summarizes the time-dependent Log_10_ reduction values and cell survival values for **1**-**3**-Chl and **1**-**3**-SnChl toward planktonic cells of *S. aureus* and *E. coli* after photoirradiation with M625L3 and M660L4 Thorlabs LEDs.

**Figure 5:**
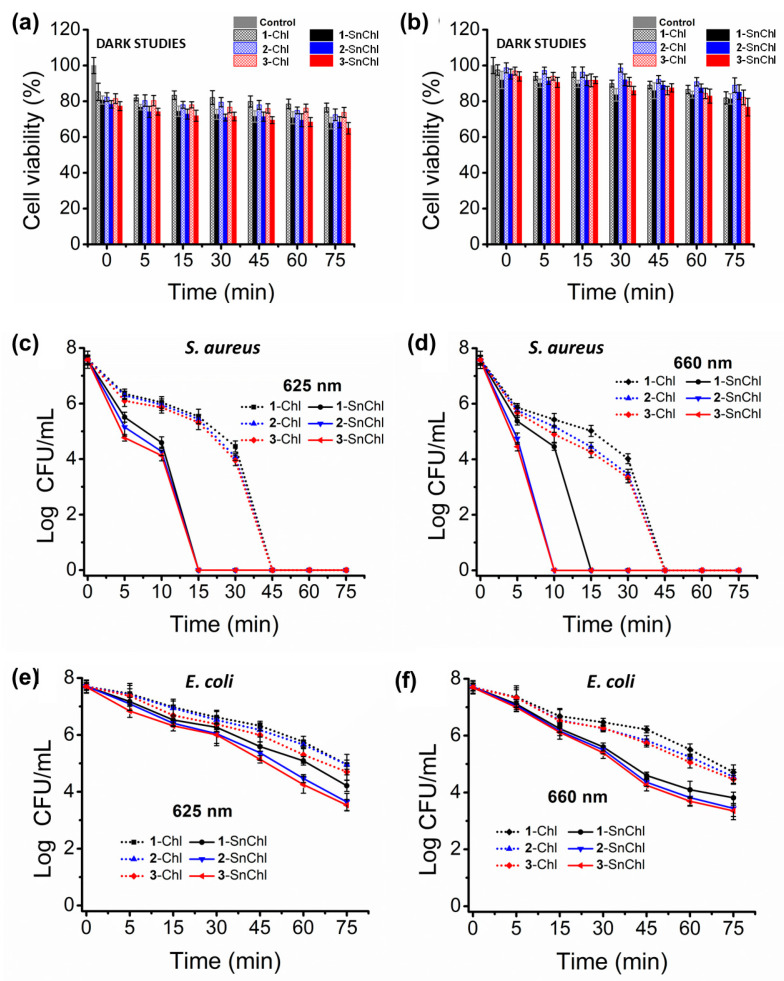
Cell viability plots for planktonic (**a**) *S. aureus* and (**b**) *E. coli* upon treatment with (**a**) 1 µM and (**b**) 5 µM of **1**-**3**-Chl and 1-**3**-SnChl in the dark. Log_10_ reduction and cell survival values with (**c**,**d**) 1 µM and (**e**,**f**) 5 µM of **1**-**3**-Chl and 1-**3**-SnChl against planktonic cells in 1% DMSO/PBS after illumination with Thorlabs M625L3 (240 mW·cm^−2^) and M660L4 (280 mW·cm^−2^) LEDs. The error bars represent the mean ± standard deviation of three independent assays each with three replicates.

**1**-**3**-Chl free-base chlorins exhibited favorable PACT activities toward planktonic *S. aureus* with high Log_10_ reduction values after 45 min illumination, while **1**-**3**-SnChl have higher PACT activities toward planktonic *S. aureus* than those of the **1**-**3**-Chl free-base ligands. This is demonstrated by the complete eradication of planktonic *S. aureus* colonies after 15 min illumination for **1**-**3**-SnChl and also 10 min for **2**-**3**-SnChl at 660 nm (Figure 5, Table 5), due to the high Φ_Δ_ values for **1**-**3**-SnChl that are related to the presence of the heavy Sn(IV) ion.

**1**-**3**-Chl free-base chlorin ligands also exhibit moderate Log_10_ reduction values under illumination at 660 nm for planktonic *E. coli* in the 2.20−2.65 range after illumination for 75 min, which denotes ≥99% cell reduction and ≤0.9 cell survival. This is in contrast to the favorable performance **1**-**3**-Chl exhibited toward planktonic *S. aureus*. This trend is anticipated in the context of neutral PS dyes toward Gram-(−) *E. coli*, since *E. coli* bacteria have a permeability barrier, which restricts PS dyes from penetrating the cells [41,42]. On the other hand, **1**-**3**-SnChl exhibited favorable PACT activities toward planktonic *E. coli* with higher Log_10_ reduction values of ≥3.50 after 75 min illumination with Thorlabs M625L3 and M660L4 LEDs (Table 5). These values are greater than the optimal ≥3 Log_10_ (99.9% cell reduction) United States Food and Drug Administration’s guideline for antimicrobial agents [43,44]. This suggests that **1**-**3**-SnChl can be efficient PS dyes for the eradication of planktonic *E. coli*. The effects of different *meso*-aryl substituents on the PACT activities of the chlorin dyes against planktonic *S. aureus* and *E. coli* are consistent with those observed for their PDT activities. The *meso*-4-hydroxyphenyl (**2**-Chl, **2**-SnChl) and -4-hydroxy-3-methoxyphenylchlorins (**3**-Chl, **3**-SnChl) have slightly higher PACT activities against *E. coli* than the *meso*-methoxyphenylchlorin (**1**-Chl, **1**-SnChl) (Figure 5, Table 5). This can be attributed to the hydrophilic vanillic and phenolic *meso*-aryl groups of the chlorin PS dyes, which can interact favorably with the planktonic *S. aureus* and *E. coli* cell wall resulting in slightly enhanced PS penetration and cellular uptake [39,45].

### 2.6. In Vitro PACT Cytotoxicity Studies against Biofilms of S. aureus and E. coli Bacteria

**Figure 6:**
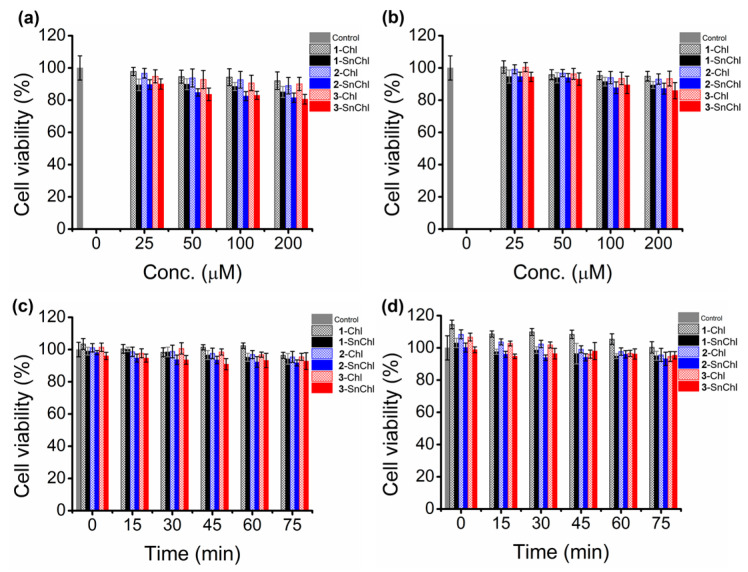
Cell viability plots of biofilm cells of (**a**,**c**) *S. aureus*, and (**b**,**d**) *E. coli* in the dark upon treatment with gradient concentrations of **1**-**3**-Chl and **1**-**3**-SnChl for 30 min with viable cells quantified by crystal violet stain, and (**c**) upon treatment with 25 µM and (**d**) 50 µM of the dyes for 75 min. The error bars represent the mean ± standard deviation of three independent assays each with three replicates.

**Figure 7:**
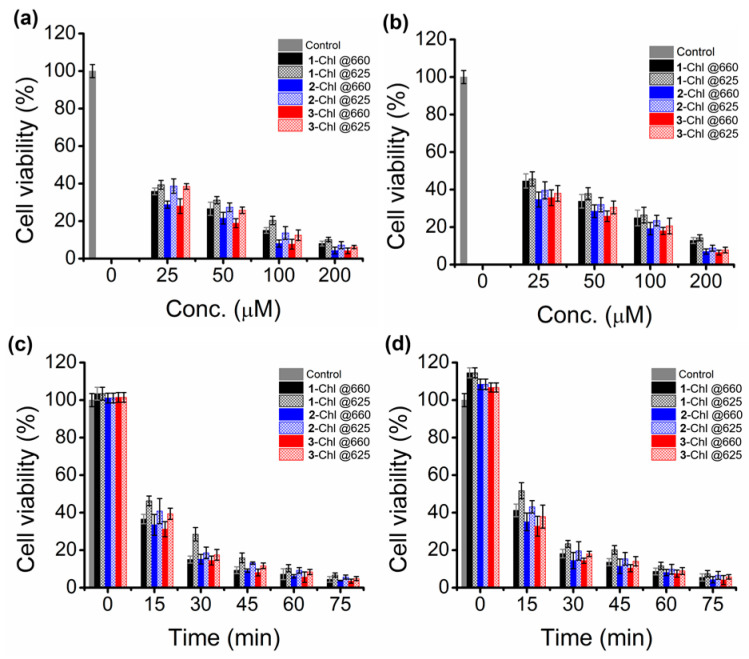
The cell viability plots for biofilm cells of (**a**) *S. aureus* and (**b**) *E. coli* upon treatment with gradient concentrations of **1**-**3**-Chl after 30 min illumination with Thorlabs M625L3 (240 mW·cm^−2^) and M660L4 (280 mW·cm^−2^) LEDs with cell quantification by crystal violet stain. (**c**,**d**) The cell viability plots of biofilm cells of (**c**) *S. aureus* and (**d**) *E. coli*, upon treatment with (**c**) 25 µM and (**d**) 50 µM of **1**-**3**-Chl after illumination with Thorlabs M625L3 (240 mW·cm^−2^) and M660L4 (240 mW·cm^−2^) LEDs with viable colonies quantified by the viable colony count method. The error bars represent the mean ± standard deviation of three independent assays each with three replicates.

**Figure 8:**
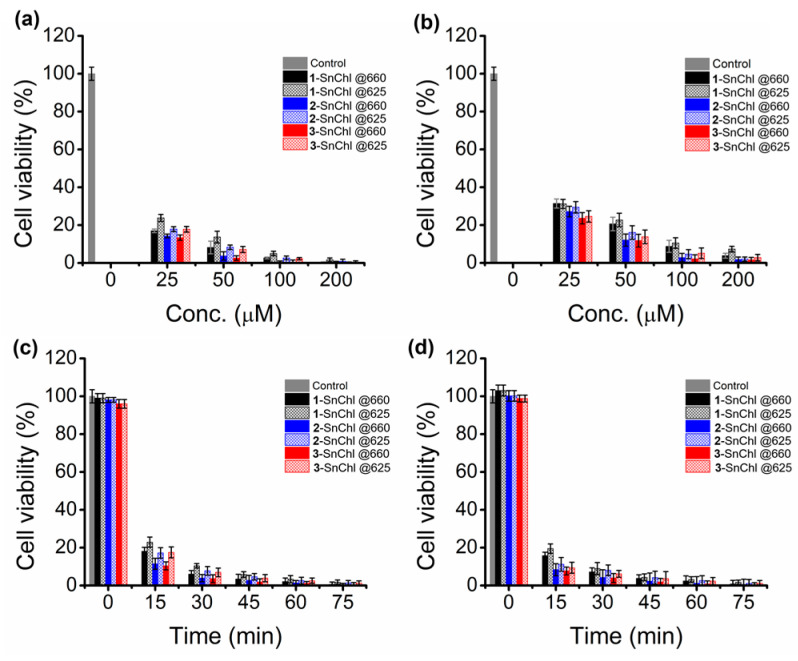
The cell viability plots for biofilm cells of (**a**) *S. aureus* and (**b**) *E. coli* upon treatment with gradient concentrations of **1**-**3**-SnChl after 30 min illumination with Thorlabs M625L3 (240 mW·cm^−2^) and M660L4 (280 mW·cm^−2^) LEDs with cell quantification by crystal violet stain. (**c**,**d**) The cell viability plots of biofilm cells of (**c**) *S. aureus* and (**d**) *E. coli*, upon treatment with (**c**) 25 µM and (**d**) 50 µM of **1**-**3**-SnChl after illumination with Thorlabs M625L3 (240 mW·cm^−2^) and M660L4 (280 mW·cm^−2^) LEDs with viable colonies quantified by the viable colony count method. The error bars represent the mean ± standard deviation of three independent assays each with three replicates.

The optimal concentrations of **1**-**3**-Chl and **1**-**3**-SnChl used during the time-dependence studies were 25 µM for *S. aureus* biofilms and 50 µM for *E. coli* biofilms (Figure 6, Figure 7 and Figure 8). The Log_10_ reduction values for *S. aureus* and *E. coli* biofilms are ≥1 Log_10_ with ≥90% cell reduction and ≤10% cell survival (Table 6). These Log_10_ reduction values are much lower than those observed against the planktonic *S. aureus* and *E. coli* bacteria. This is normally anticipated in the context of *S. aureus* and *E. coli* biofilms, since biofilms are less susceptible to antimicrobial agents due to their self-securing polymeric matrix, which restricts drug penetration [46,47,48]. The photoexcitation of chlorin dyes at 660 nm resulted in slightly higher Log_10_ reduction values for *S. aureus* and *E. coli* biofilms than those obtained with 625 nm irradiation (Table 6). This observation agrees with previous reports, which explained that NIR illumination facilitates deeper penetration of light into the tissue resulting in enhanced PACT activities [16,36,37]. 

The *meso*-4-hydroxyphenyl and -4-hydroxy-3-methoxyphenylchlorins (**2**-**3**-Chl, **2**-**3**-SnChl) exhibited slightly higher PACT activities toward biofilm cells of *S. aureus* and *E. coli* than the *meso*-methoxyphenylchlorins (**1**-Chl, **1**-SnChl) (Table 6). This could be associated with favorable interactions between the hydrophilic vanillic and phenolic groups of the chlorin dyes and the biofilm cells, resulting in slightly enhanced drug penetration and cellular uptake [45]. As anticipated based on their high Φ_Δ_ values, the Log_10_ reduction values for **1**-**3**-SnChl are slightly higher toward biofilm cells of *S. aureus* and *E. coli* in contrast to the **1**-**3**-Chl free-base ligands (Figure 7 and Figure 8, Table 6).

## 3. Discussion

The PDT activities of **1**-**3**-Chl and **1**-**3**-SnChl are broadly similar to those of other tetraarylchlorins with thien-2-yl and methylthiophenyl rings that we have reported previously using a similar approach with a Thorlabs M660L4 LED (Table 7) [15,16,17]. In a similar manner, the PI values for **1**-**3**-SnChl are significantly larger than those for **1**-**3**-Chl, demonstrating again that coordination of a Sn(IV) ion enhances the PDT activities. This may be related to their higher Φ_Δ_ values (Table 2) and scope for a decrease in aggregation due to *trans*-axial ligation [10]. In a similar manner, the PACT activity properties of **1**-**3**-Chl and **1**-**3**-SnChl against planktonic *S. aureus* and *E. coli* (Table 8) are broadly similar to those reported previously for dyes with thien-2-yl and methylthiophenyl *meso*-aryl rings [15,16,17]. The Log_10_ reduction values corresponding to ≈0% cell survival were achieved more quickly in the context of Gram-(+) *S. aureus* and at lower concentration than was the case previously with Sn(IV) tetramethylthiophenylchlorin [17], while moderately high Log_10_ reduction values > 3.0 were obtained for **1**-**3**-SnChl against *E. coli* (Table 8). Values > 3.0 are consistent with the FDA’s guidelines for an effective antibacterial agent [43,44]. In contrast with Sn(IV) tetramethylthiophenylchlorin [17]; however, 0% cell survival was not achieved against Gram-(−) *E. coli* for Sn(IV) complexes **1**-**3**-SnChl with 3-methoxy-, 4-hydroxy- and 3-methoxy-4-hydroxyphenyl *meso*-aryl rings. These results demonstrate that Sn(IV) tetraarylchlorin complexes merit further in depth study for use as PS dyes for PDT and against both Gram-(+) and Gram-(−) bacteria in the context of PACT.

## 4. Materials and Methods

### 4.1. Materials

All reagents were obtained commercially and used with no further purification, unless otherwise stated. Reagent-grade SnCl_2_∙2H_2_O, p-chloranil, p-toluenesulfonyl hydrazide, 1,3-diphenylisobenzofuran (DPBF), anhydrous sodium sulfate, K_2_CO_3_ and 5,10,15,20-tetraphenyl-21H,23H-porphine zinc (ZnTPP), Rose Bengal, CDCl_3_ and an MTT assay kit were purchased from Sigma Aldrich (St. Louis, MO, USA). CHCl_3_, dimethylformamide (DMF) and methanol were procured from Merck. Type II water was prepared with an Elga Purelab Chorus 2 (RO/DI) system. MCF-7 cell cultures were purchased from Cellonex^®^, while tissue-culture-grade penicillin-streptomycin-amphotericin B mix, 100 units·mL^−1^ penicillin, 100 µg·mL^−1^ streptomycin-amphotericin B and heat-inactivated fetal calf serum (FCS) were obtained from Biowest^®^. Dulbecco’s modified Eagle’s medium (DMEM) and phosphate-buffered saline (DPBS) were supplied by Lonza^®^.

### 4.2. Instrumentation

^1^H NMR spectra were measured with Bruker Avance II^TM^ 600 MHz and AMX 400 MHz instruments with the solvent residual as the internal reference (δ = 7.26 ppm for CDCl_3_). ACS spectral grade solvents were used for spectroscopic measurements. UV-visible spectra were recorded with a Shimadzu UV-2550 spectrophotometer and an Evolution 350-UV-Vis spectrophotometer from Thermo Fischer Scientific. Mass spectrometry data were measured on a Bruker AutoFLEX III Smartbeam TOF/TOF mass instrument in the positive ion mode with α-cyano-4-hydroxycinnamic acid as the MALDI matrix. Fluorescence spectra were measured with a Varian Eclipse^®^ spectrofluorometer with the optical density at the B band maxima adjusted to ~0.05. Φ_F_ values were determined in DMSO with a comparative method by using ZnTPP as the standard (Φ_F_ = 0.039 in DMSO [49]). τ_F_ values were determined with a Picoquant FluoTime 300 TCSPC setup. Decay curves were measured at the emission band maxima and deconvoluted using the Picoquant FluoFit software package version 4.6.6.0. Laser flash photolysis in DMSO was used to determine the τ_T_ values at 500 nm with an Edinburgh Instruments LP980 instrument by fitting an exponential to the decay curve. An Ekspla NT-342B laser (2.0 mJ, 7 ns and 20 Hz) provided a probe beam of ca. 430 nm at the B band maxima. The Φ_Δ_ values were determined using a comparative method in DMSO with DPBF as the scavenger and Rose Bengal as a standard (Φ_Δ_ = 0.76 in DMSO [50]). An Ekspla NT-342B laser with an OPO provided monochromatic light at a spectral crossover between the standard and the sample. Photostability tests were carried out in a 1 × 1 cm quartz cuvette, and the solutions were irradiated with a 660 nm (280 mW·cm^−^^2^) Thorlabs LED lamp mounted onto a Modulight 7710-680 medical laser system housing in a similar manner to the PDT and PACT activity measurements. The 1% DMSO/H_2_O solutions were prepared in the dark by dissolving 1 mg of sample in 6 mL of solvent. Photobleaching of the dyes at the B band maxima was determined by UV-visible absorption spectroscopy. The percentages of the initial B band intensities observed after 30 min of photoirradiation are provided in Table 2.

### 4.3. Synthesis of Free-Base Chlorins

**1**-**3**-Por and **2**-Chl have been reported previously and were prepared according to literature procedures [23,24]. **1**-Chl was synthesized following a procedure described in the literature [25] (Scheme 1). 5,10,15,20-*tetrakis*(3-methoxyphenyl)chlorin (**1**-Chl) was synthesized in this study using **1**-Por (74 mg, 1 mmol) dissolved in dry pyridine (40 mL), and K_2_CO_3_ (8 mmol) was added and stirred. The mixture was then refluxed in an inert nitrogen environment, and the same amount of *p*-toluenesulfonyl hydrazide (4 mmol) was added at 3 h intervals for 12 h. The reaction progress was monitored regularly using TLC and UV-visible absorption spectroscopy and stopped when porphyrin peaks disappeared, and the reduced bacteriochlorin peak emerged at ca. 730 nm. The reaction mixture was cooled to room temperature, extracted with CHCl_3_, washed three times with 0.1 M HCl solution, and lastly with Millipore water to remove pyridine and salts. The organic layer was dried over anhydrous sodium sulfate, and *p*-chloranil was added until the band at 730 nm disappeared. The mixture was filtered, and the organic solvent was evaporated. The crude product was purified by using silica gel chromatography with 3:1 CH_2_Cl_2_/petroleum ether eluent to afford the **1**-Chl target compound as a purple solid. Yield: 31 mg (43%). **1**-Chl (C_48_H_40_N_4_O_4_): ^1^H NMR (400 MHz, CDCl_3_) δ 8.62 (d, J = 4.7 Hz, 2H), 8.46 (d, J = 3.9 Hz, 2H), 8.23 (d, J = 4.7 Hz, 2H), 7.71 (d, J = 7.6 Hz, 4H), 7.55 (d, J = 4.0 Hz, 2H), 7.35 (d, J = 2.3 Hz, 2H), 7.33 (d, J = 2.3 Hz, 2H), 7.22 (d, J = 2.3 Hz, 3H), 7.20 (d, J = 2.6 Hz, 3H), 4.19 (s, 4H), 3.94 (s, 12H), −1.48 (s, 2H). UV-vis (DMSO): λ_max_ nm (log ε) 420 (5.07), 515 (3.77), 549 (3.51), 596 (3.22), 651 (4.00). MS (MALDI TOF): Calcd *m/z* 736.30; observed: 737.43 [M+H]^+^.

**3**-Chl was synthesized in a similar manner to that described for **1**-Chl (Scheme 1). 5,10,15,20-Tetra(4-hydroxy-3-methoxyphenyl)chlorin **3**-Chl was obtained in 35% yield. **3**-Chl (C_44_H_32_N_4_O_4_): ^1^H NMR (400 MHz, DMSO-*d*_6_) δ 9.52 (s, 4H), 8.66 (d, J = 5.1 Hz, 2H), 8.38 (s, 2H), 8.25 (d, J = 5.1 Hz, 2H), 7.58 (d, J = 8.2 Hz, 4H), 7.51 (d, J = 8.3 Hz, 4H), 7.14 (d, J = 4.2 Hz, 4H), 4.20 (s, 4H), 3.83 (d, J = 7.7 Hz, 12H), −1.57 (s, 2H).UV-vis (DMSO): λ_max_ nm (log ε) 428 (5.05), 520 (3.93), 560 (3.83), 601 (3.93), 653 (4.01). MS (MALDI TOF): Calcd *m/z* 800.28; observed: 801.43 [M+H]^+^.

### 4.4. Synthesis of Sn(IV) Chlorins

For the preparation of dichlorotin(IV) 5,10,15,20-tetra(3-methoxyphenyl)chlorin, **1**-Chl (45 mg, 0.06 mmol) was dissolved in 15 mL dry CHCl_3_/methanol solution (1:1 *v*/*v*) and warmed. SnCl_2_·2H_2_O (98 mg, 0.43 mmol) was added, and the reaction mixture was refluxed for 12 h (Scheme 1). The reaction progress was monitored using TLC and UV-visible absorption spectroscopy. The reaction mixture was cooled to room temperature, and organic solvents were evaporated. The residual crude product was purified on a neutral alumina column chromatography and CH_2_Cl_2_/MeOH (7:1 *v*/*v*) eluent to afford the target compound **1**-SnChl as green crystals. Yield: 77%. **1**-SnChl (C_48_H_38_Cl_2_N_4_O_4_Sn): ^1^H NMR (400 MHz, CDCl_3_) δ 8.65 (d, J = 5.1 Hz, 2H), 8.57 (d, J = 9.0 Hz, 2H), 8.20 (d, J = 5.2 Hz, 2H), 7.81 (d, J = 8.0 Hz, 2H), 7.58 (d, J = 8.1 Hz, 4H), 7.45 (d, J = 7.3 Hz, 4H), 7.16 (d, J = 6.7 Hz, 2H), 7.10 (d, J = 9.7 Hz, 2H), 4.37 (s, 4H), 3.95 (s, 12H).UV-vis (DMSO): λ_max_ nm (log ε) 430 (5.23), 564 (3.96), 602 (4.03), 627 (4.12). MS (MALDI TOF): Calcd *m/z* for [M−2Cl]^+^ = 924.13, [M−Cl]^+^ = 889.16; observed: 889.16 [M−Cl]^+^. 

**2**-**3**-SnChl Sn(IV) chlorin complexes were synthesized in a similar manner to the procedure described for **1**-SnChl (Scheme 1).

Dichlorotin(IV) 5,10,15,20-tetra(4-hydroxyphenyl)chlorin (**2**-SnChl) was obtained in 65% yield. **2**-SnChl (C_44_H_30_Cl_2_N_4_O_4_Sn): ^1^H NMR (400 MHz, DMSO-*d*_6_) δ 9.05 (s, 2H), 8.84 (s, 2H), 8.38 (d, J = 4.9 Hz, 2H), 8.18 (d, J = 4.3 Hz, 2H), 8.13 (d, J = 7.5 Hz, 2H), 8.07 (s, 2H), 8.00 (d, J = 5.2 Hz, 2H), 7.96 (d, J = 2.8 Hz, 2H), 7.64 (d, J = 8.8 Hz, 4H), 7.23 (d, J = 6.3 Hz, 2H), 7.17 (d, J = 8.6 Hz, 2H), 7.11 (d, J = 8.2 Hz, 2H), 4.14 (s, 4H). UV-vis (DMSO): λ_max_ nm (log ε) 438 (5.11), 576 (3.79), 613 (3.99), 629 (4.16). MS (MALDI TOF): Calcd *m/z* for [M−2Cl]^+^ = 868.07, [M−Cl]^+^ = 833.09; observed: 834.11 [M−Cl+H]^+^.

Dichlorotin(IV) 5,10,15,20-tetra(4-hydroxy-3-methoxyphenyl)chlorin (**3**-SnChl) was obtained in 72% yield. **3**-SnChl (C_48_H_38_Cl_2_N_4_O_8_Sn): ^1^H NMR (400 MHz, DMSO-*d*_6_) δ 9.68 (s, 4H), 8.71 (d, J = 4.7 Hz, 2H), 8.22 (d, J = 5.1 Hz, 2H), 8.04 (d, J = 10.4 Hz, 2H), 7.79 (d, J = 9.6 Hz, 4H), 7.68 (d, J = 14.2 Hz, 4H), 7.13 (d, J = 8.7 Hz, 4H), 4.19 (s, 4H), 3.94 (s, 12H). UV-vis (DMSO): λ_max_ nm (log ε) 439 (5.05), 575 (3.95), 615 (4.19), 630 (4.36). MS (MALDI TOF): Calcd *m/z* for [M−2Cl]^+^ = 988.11, [M−Cl]^+^ = 953.14; observed: 953.48 [M−Cl]^+^.

### 4.5. PDT Studies

MCF-7 cells were cultured as previously described [51] in DMEM containing 4.5 g·L^−1^ glucose with L-glutamine and phenol red. The media was supplemented with 10% (*v*/*v*) heat-inactivated FCS and 5% 100 unit mL^−1^ penicillin 100 µg·mL^−1^ streptomycin amphotericin B. The cells were grown in a T75 cm^2^ vented flask (Porvair) and incubated in a humidified 5% CO_2_ atmosphere at 37 °C until 80% confluence was achieved. The cells were rinsed with DPBS before routine standard trypsinization during subculturing and cell harvesting. The trypsinized cells were treated with trypan blue dye exclusion assay (0.40% trypan blue solution), and a hemocytometer was used to count viable cells. Cells were seeded at a density of 10,000 cells/well in 96-well plates in supplemented red-phenol DMEM medium and incubated under a humidified 5% CO_2_ for 24 h at 37 °C to enable cell attachment to the wells. The attached cells were then rinsed twice using 100 µL DPBS, and subsequently, 100 µL of the drugs (the studied compounds) in the DMEM medium were administered over gradient concentration ranges. The drug stock solutions were prepared in DMSO since they are insoluble in aqueous media. After dilution, appropriate drug aliquot volumes were prepared in DMEM media such that the highest gradient concentrations consisted of <1% (*v*/*v*) DMSO. The control cells were supplemented with DMEM medium alone, with the effect of DMSO on the cells investigated by incubating the cells in 1% (*v*/*v*) DMSO-DMEM media. The plates were then re-incubated for 24 h in the dark for further studies that include in vitro dark cytotoxicity and PDT activity studies.

The MCF-7 cancer cells treated with the different drug concentrations were rinsed thrice with 100 µL DPBS to remove any residual drug. DMEM media was added with no red phenol. For in vitro dark cytotoxicity studies, the drug-treated cells were not photoirradiated, while for light studies, the treated cells were photoirradiated with a M625L3 or M660L4 Thorlabs LED mounted onto a Modulight 7710-680 medical laser system housing. The excitations of the PS dyes were performed at the red end of the visible since the dyes used in this work are intended for use in the treatment of deep-seated infected soft tissues and tumors [52,53]. Both Thorlabs M625L3 and M660L4 LEDs were used for 20 min photoirradiations of cells treated with **1**-**3**-Chl and **1**-**3**-SnChl. The DMEM was replaced with red phenol, and the cells were incubated further for 24 h. Cell viabilities were determined using the MTT assay protocols following the manufacturer’s specifications by measuring the absorbances of the assays at 540 nm on a Synergy 2 multi-mode microplate reader (BioTek^®^). All experiments were carried out in triplicate, with the data analyzed statistically using Student’s *t*-test and ANOVA. The percentage cell viabilities were calculated as the percentage ratio of the absorbance of the drug-treated cells against the untreated controls [51], as described in Equation (1).

(1) $$\%\;\mathrm{Cell}\;\mathrm{Viability}=\frac{\;\mathrm{Absorbance}\;\mathrm{of}\;\mathrm{samples}\;\mathrm{at}\;540\;\mathrm{nm}}{\mathrm{Absorbance}\;\mathrm{of}\;\mathrm{control}\;\mathrm{at}\;540\;\mathrm{nm}}\times 100$$

Since the IC_50_ value is another important parameter for determining drug efficacy, it was calculated for the dyes by nonlinear regression analysis using GraphPad Prism 5. During in vitro PDT studies, the IC_50_ value provides a half-maximal inhibitory concentration of a PS dye for inhibiting the growth or killing 50% of cancer cells after drug incubation and light treatment. 

### 4.6. Lipophilicity Studies

The lipophilicity tests were carried out in triplicate for each dye complex studied by using the “shake-flask” method [54]. The sample solutions were prepared using 0.5 mg of each dye complex dissolved in 10 mL of dry CHCI_3_. For each 3 mL CHCl_3_ solution of the complex, the absorbance at the Soret band maxima (A_o_) was measured. Then, 3 mL of Millipore water was added to each 3 mL CHCl_3_ solution, followed by stirring of the mixture at room temperature for 4 h. Thereafter, the mixture was centrifuged at 5000 rpm for 10 min to allow the separation of the water and CHCl_3_ phases. Then, the absorbance of the CHCl_3_ layer (A_f_) was measured for each complex mixture, with the value for the water phase determined from the difference between the A_o_ and A_f_ values of the dye complex in CHCl_3_. The partition coefficient values (Log P_o/w_) for the complexes in CHCl_3_:H_2_O were derived using Equation (2) [54].

P_octanol_ = [1.343 + Log P_chloroform_]/1.126 (2)

### 4.7. PACT Studies

The PACT activities of the PS dyes were carried out against planktonic and biofilms cells of Gram-(+) *S. aureus* (ATCC^®^ 25923^TM^) and Gram-(−) *E. coli* bacteria (ATCC^®^ 25922^TM^) strains. Photoinactivation experiments were performed as described in the literature with slight modifications [55,56,57]. The viable bacteria colonies were estimated using the direct viable colony count and by an indirect method with the crystal violet staining assay. The planktonic bacteria cells were estimated using the direct method only by counting the viable colonies formed on the agar plates using a bacteria counter, as described previously in the literature [55,56,57], while for biofilms cells, both the viable colony count and indirect methods were used [35,58,59,60]. The biofilm biomass was quantified indirectly via the crystal violet assay by measuring the absorbance intensity at 590 nm on a Synergy 2 multi-diode microplate reader (BioTek). The PS dye drug stock solutions were first prepared in DMSO since they were insoluble in PBS alone with the appropriate drug aliquots volumes prepared to 1% (*v*/*v*) DMSO-PBS solution after serial dilution. Photoinactivation experiments were carried out in the dark, and after photoirradiation with an M625L3 or M660L4 Thorlabs LED mounted onto a Modulight 7710-680 medical laser system housing. For all in vitro dark cytotoxicity and photoinactivation activity studies of the dyes against both the planktonic and biofilm cells of *S. aureus* and *E. coli* bacteria strains, the effect of PS dye drug concentrations at a fixed fluence (light) dosage and the effect of different irradiation times at a fixed drug dye concentration were investigated.

#### 4.7.1. Planktonic Bacteria

*S. aureus* and *E. coli* bacteria strains were grown anaerobically on agar plates to obtain colonies of each by following the manufacturer’s specifications. A single colony of each strain to be studied was inoculated into a 5 mL freshly prepared Lura nutrient broth. The culture mixture was vortexed and incubated at 37 °C with agitation (*ca.* 200 rpm) in a rotary shaker incubator for 18 and 48−72 h for *S. aureus* and *E. coli*, respectively. Aliquots from the bacteria culture mixtures were taken regularly to measure their optical densities until the bacterial growth reached a mid-logarithmic phase (OD 620 nm, 0.6−0.8). The bacteria pellets were then harvested through centrifugation for 15 min at 3000 rpm and washed three times with PBS to remove residual nutrient broth. The bacteria pellets were re-suspended in 4 mL PBS and further diluted to 1:1000 (*v*/*v*) in PBS to obtain the working bacteria culture stock solutions. The viable colonies count of the freshly prepared *S. aureus* and *E. coli* bacteria cultures were determined by serial dilution of the bacteria culture stock solutions by factors of 10^−4^, 10^−5^, 10^−6^, 10^−7^, 10^−8^, and 10^−9^. A 100 µL aliquot of each sample solution was aseptically inoculated on the agar plates in triplicate and incubated at 37 °C for 24 h to determine the optimum bacteria count. The viable bacteria colonies were counted on a Scan 500^®^ series Automatic Colony Counter. Optimized bacteria solutions of colony-forming units (CFU/mL) ranging from 2.78 to 3.01 × 10^8^ and 1.62 to 2.01 × 10^8^ CFU/mL for *S. aureus* and *E. coli*, respectively, were used in the subsequent studies.

The experimental procedure followed for sample preparation for in vitro dark and light studies of the dyes for photoinactivation of planktonic *S. aureus* and *E. coli* bacteria strains was the same. For time-dependence studies, a 5 mL bacteria suspension (10^8^ CFU/mL) of *S. aureus* or *E. coli* bacteria strains in 1% DMSO/PBS solution was incubated with appropriate concentrations of the porphyrinoid dye drugs at 37 °C in a rotary shaking incubator at 200 rpm for 30 min in the dark. Half (2.5 mL) of the drug-incubated bacteria suspension was transferred into a 24-well plate for light studies, and the remaining half (2.5 mL) was kept in another 24-well plate in the dark for in vitro dark cytotoxicity studies. 

During light studies, the drug-treated bacteria suspensions were irradiated with M625L3 or M660L4 Thorlabs LEDs mounted onto a Modulight 7710-680 medical laser system housing over different time intervals. For **1**-**3**-Chl and **1**-**3**-SnChl, 1 µM of the dye was administered against *S. aureus* and 5 µM against *E. coli* bacteria suspensions. For the bacteria suspensions treated with **1**-**3**-Chl and **1**-**3**-SnChl, both Thorlabs M625L3 and M660L4 LEDs were used for photoirradiation at 5, 10, 15, 30, 45, 60, and 75 min intervals. After light and dark treatments at the considered time intervals, a 100 µL aliquot of the samples was aseptically inoculated on the agar plate, which was incubated for 18 h at 37 °C. Viable bacteria colonies were counted with a Scan 500^®^ series Automatic Colony Counter to determine colony-forming unit (CFU/mL) values. The controls were *S. aureus* and *E. coli* bacteria suspensions (colony-forming units ≈ 10^8^ CFU/mL) with no PS dye drug. The cell survival fractions were calculated by comparing the drug-treated bacteria with the control. The Log_10_ reductions were calculated using Equation (3):

Log reduction = Log (A) − Log (B)(3)

A and B are the number of viable colonies (CFU/mL) of bacteria for the untreated and treated samples, respectively. All the experiments were carried out in three independent triplicates, and data were analyzed statistically using student *t*-test and ANOVA. Prior to time-dependence studies, the effect of gradient concentrations of the dyes was analyzed at fixed light intensity (fluence) to establish the optimal concentrations for study. For bacteria suspensions treated with **1**-**3**-Chl and **1**-**3**-SnChl, gradient concentrations of 0.5, 1, 1.5, 2, 2.5, and 5 µM were administered against *S. aureus*, and 1, 2.5, 5, 10, and 15 µM against *E. coli* with photoirradiation carried out for 45 min using a Thorlabs M625L3 LED mounted onto a Modulight 7710-680 medical laser system housing.

#### 4.7.2. Biofilm Bacteria

The freshly prepared planktonic *S. aureus* and *E. coli* bacteria cultures were diluted to 1 × 10^6^ CFU/mL in Tryptic Soy Broth (TBS). The TBS-supplemented bacteria cultures (200 µL/well) were seeded into the 96-well plates and incubated anaerobically under static conditions at 37 °C with 5% CO_2_ for 24 and 72 h for *S. aureus* and *E. coli*, respectively, to form *S. aureus* and *E. coli* single-species bacteria biofilms. The wells with media alone provided the negative controls. After incubation, the culture media was discarded, and the wells were rinsed gently twice with PBS to remove the residual TBS and the non-adherent planktonic bacteria cells. The plates were air-dried at room temperature for 30 min to fix the adherent cells. 

The biofilm bacteria biomass was quantified using the crystal violet assay. First, 150 µL/well of an aliquot of 0.1% (*w*/*v*) of crystal violet was used to stain the biofilm-coated wells for 15 min at room temperature. The crystal violet was then discarded, and the wells were washed three times using 200 µL PBS/well to remove the residual dye. 150 µL/well of PBS was again added to the stained wells, and the absorbance of well solutions was measured at 590 nm on a Synergy 2 multi-mode microplate reader (BioTek^®^). All experiments were carried out in triplicate, and the Z-score method was used to identify outlier values. Non-outlier values were used to calculate the average biofilm formed. The average negative control value was subtracted from the obtained biofilms absorbance data, and the data were provided as mean absorbance ± standard deviation. Biofilms were considered to have formed when the absorbance value at 590 nm was three times the standard deviation of the negative control mean absorbance [61]. All experimental data were analyzed statistically using a student *t*-test and ANOVA. Viable cell counts for the single species *S. aureus* and *E. coli* biofilms were also estimated by the viable colony count method. The biofilms formed in the 96-well plates were solubilized by adding 200 µL/well of PBS and sonicating the plates vigorously for 10 min, followed by scraping of the wells to dislodge the adherent cells. The resultant well suspension was vortexed to homogenize the solution and serial diluted 10-fold times, and a 100 µL aliquot of the diluted sample was then aseptically inoculated on the agar plate in triplicates followed by incubation at 37 °C for 18 h. The viable colonies count (CFU/mL) was carried out on a Scan 500^®^ series Automatic Colony Counter. All the experimental data were analyzed statistically with a student *t*-test and ANOVA.

Initially, the effect of the various dye concentrations on inhibiting the growth of the single-species biofilms of *S. aureus* and *E. coli* strains experiments were carried out. For these concentration studies, the single species biofilms of *S. aureus* and *E. coli* strains were incubated with 100 µL/well aliquots of the dye series at various concentrations for 30 min. During the light studies, the drug-incubated 96-well plate biofilms were photoirradiated with a 625 or 660 nm Thorlabs LED for 30 min, while for the dark studies, the drug-treated plates were kept in the dark (in the absence of irradiation) for 30 min to determine the dark cytotoxicities of the drugs. For **1**-**3**-Chl and **1**-**3**-SnChl, the single-species *S. aureus* and *E. coli* biofilms were treated with drug concentrations of 25, 50, 100 and 200 µM with both M625L3 and M660L4 Thorlabs LEDs. The controls were the biofilm-coated wells with PBS media alone (untreated biofilms). After the dark and light treatments, the supernatant liquid was discarded, and the wells were gently rinsed with PBS. The biofilm cell densities were then quantified using crystal violet assay by measuring the absorbance of the assay at 590 nm on a Synergy 2 multi-mode microplate reader (BioTek^®^), and also by using the viable colony count method as previously described in Section 4.7.1. For time-dependence studies, **1**-**3**-Chl and **1**-**3**-SnChl, 25 and 50 µM of the dyes were administered against *S. aureus* and *E. coli* biofilms, respectively. Both Thorlabs M625L3 and M660L4 LEDs were used for the photoirradiations. The ability of the dyes to inhibit the single species *S. aureus* and *E. coli* biofilms was quantified using the crystal violet assay and viable colonies count methods as previously described, and the data were compared with the controls. All experiments were performed with three independent triplicates and compared with the control, and data were analyzed statistically with Student’s *t*-test and ANOVA.

## 5. Conclusions

The photoexcitation of chlorin dyes with 3-methoxy-, 4-hydroxy- and 3-methoxy-4-hydroxyphenyl *meso*-aryl rings (**1**-**3**-Chl) at the red end of the electromagnetic spectrum results in favorable PDT activities against MCF-7 breast cancer cells and PACT activities against Gram-(+) *S. aureus* and Gram-(−) *E. coli* bacterial strains. Sn(IV) chlorin complexes **1**-**3**-SnChl have more favorable PDT and PACT activities than the corresponding free base **1**-**3**-Chl dyes. This is probably due to their high singlet oxygen quantum yields and decreased aggregation due to *trans*-axial ligation, which favorably enhances the PDT and PACT activities of the chlorin dyes. The data demonstrate that readily synthesized tetraarylchlorins and their metal complexes merit further in-depth study for use as PS dyes in biomedical applications. It is particularly noteworthy that high Log_10_ reduction values >3.0 were obtained for **1**-**3**-SnChl against Gram-(−) *E. coli* despite the absence of cationic charges in the structures. Data of this type have only previously been reported for tetrarylchlorins and their Sn(IV) complexes [10,15,17] with sulfur-atom-containing thien-2-yl, 5-bromothien-2-yl, tetramethylthiophenyl *meso*-aryl rings.

## Figures and Tables

**Table 1:** Absorption and emission bands of **5**-**8**-Chl and **1**-**3**-SnChl in DMSO.

Sample	λ_Abs_ (nm)		λ_Em_ (nm)
	B Band	Q Bands	
**1**-Chl	420	515, 549, 596, 651	655, 717
**2**-Chl	426	524, 556, 599, 650	656, 721
**3**-Chl	428	520, 560, 601, 663	658, 724
**1**-SnChl	430	564, 602, 627	630, 679
**2**-SnChl	438	576, 613, 629	639, 698
**3**-SnChl	439	575, 615, 630	641, 704

**Table 2:** Photophysicochemical and photostability properties of **1**-**3**-Chl and **1**-**3**-SnChl in DMSO.

Sample	Φ_F_	τ_F_ (ns)	τ_T_ (μs)	Φ_Δ_	Photostability ^a^
	±0.020	±0.01	±1	±0.01	(%)
**1**-Chl	0.084	6.56	188	0.59	67
**2**-Chl	0.098	5.76	189	0.58	64
**3**-Chl	0.074	5.60	111	0.62	63
**1**-SnChl	0.018	0.67	87	0.71	94
**2**-SnChl	0.015	0.75	85	0.70	96
**3**-SnChl	0.014	0.35	96	0.69	90

^a^ Intensity of the B band after 30 min photoirradiation relative to its initial intensity.

**Table 3:** IC_50_, PI, and percentage cell viability values for **1**-**3**-Chl and **1**-**3**-SnChl (at 25 μM) against MCF-7 cancer cells.

	IC_50_ (μM)			PI ^c^		Cell Viability (%) at 25 µM		
	Dark ^a^	Light ^b^				Dark	Light ^b^	
		625 nm LED	660 nm LED	625 nm LED	660 nm LED		625 nm LED	660 nm LED
**1**-Chl	>25	22.4 (±1.3)	13.9 (±1.2)	1.5	2.0	85.9 (±4.8)	49.8 (±5.3)	41.9 (±3.6)
**2**-Chl	>25	15.5 (±1.2)	9.4 (±1.1)	2.1	2.7	80.7 (±9.1)	46.4 (±4.4)	35.8 (±4.1)
**3**-Chl	>25	10.8 (±0.9)	6.1 (±0.8)	2.3	4.1	75.8 (±5.2)	38.7 (±5.7)	28.1 (±5.1)
**1**-SnChl	>25	9.4 (±1.1)	4.1 (±0.9)	3.4	16.7	82.4 (±2.2)	26.9 (±6.6)	11.1 (±4.8)
**2**-SnChl	>25	6.3 (±1.0)	2.2 (±0.8)	5.8	22.7	69.7 (±2.6)	8.7 (±1.6)	7.9 (±1.2)
**3**-SnChl	>25	3.8 (±0.6)	1.1 (±0.3)	6.6	24.8	81.5 (±3.4)	7.6 (±2.2)	3.9 (±1.2)

^a^ Twenty-four-hour incubation dark cytotoxicity data and ^b^ phototoxicity data against MCF-7 cancer cell line upon exposure to Thorlabs M625L3 (240 mW·cm^−2^, 20 min) and M660L4 (280 mW·cm^−2^, 20 min) LEDs. ^c^ PI = phototoxicity index (dark and light IC_50_ values toxicity ratio).

**Table 4:** Water partition coefficient values (Log P_o/w_) of **1**-**3**-SnChl.

Sample	Log P_o/w_
**1**-SnChl	1.39
**2**-SnChl	0.96
**3**-SnChl	1.09

**Table 5:** Log reduction and cell survival values for **1**-**3**-Chl and **1**-**3**-SnChl against planktonic cells of *S. aureus* and *E. coli* in 1% DMSO/PBS after illumination with Thorlabs M625L3 (240 mW·cm^−2^) and M660L4 (280 mW·cm^−2^) LEDs.

	*S. aureus*		*E. coli*			
	625 nm LED	660 nm LED	625 nm LED		660 nm LED	
	Log Reduction ^a^		Log Reduction ^a^	Cell Survival (%)	Log Reduction ^a^	Cell Survival (%)
**1**-Chl	7.67 @45 min	7.67 @45 min	1.96	1.10	2.20	0.62
**2**-Chl	7.67 @45 min	7.67 @45 min	2.10	0.87	2.47	0.32
**3**-Chl	7.67 @45 min	7.67 @45 min	2.30	0.50	2.65	0.22
**1**-SnChl	7.67 @15 min	7.67 @15 min	3.50	0.03	3.90	0.01
**2**-SnChl	7.67 @15 min	7.67 @10 min	3.72	0.02	4.24	0.01
**3**-SnChl	7.67 @15 min	7.67 @10 min	3.87	0.01	4.36	<0.01

^a^ Illumination time in minutes required to achieve a Log_10_ reduction value corresponding to 0% cell viability is provided after the @ character. Otherwise, the Log10 reduction value at 75 min is provided.

**Table 6:** Log reduction and cell survival values for chlorin dyes against biofilm cells of *S. aureus* and *E. coli* in 1% DMSO/PBS after illumination for 75 min with Thorlabs M625L3 (240 mW·cm^−2^) and M660L4 (280 mW·cm^−2^) LEDs.

	*S. aureus* Biofilm Cells				*E. coli* Biofilm Cells			
	625 nm		660 nm		625 nm		660 nm	
	Log Reduction	Cell Survival (%)	Log Reduction	Cell Survival (%)	Log Reduction	Cell Survival (%)	Log Reduction	Cell Survival (%)
**1**-Chl	1.20	6.7	1.30	4.5	1.13	7.5	1.26	5.5
**2**-Chl	1.25	5.7	1.42	3.8	1.18	6.6	1.37	4.3
**3**-Chl	1.30	4.8	1.46	3.4	1.23	5.8	1.38	4.1
**1**-SnChl	1.76	1.7	2.20	0.6	1.77	1.7	2.10	0.9
**2**-SnChl	1.86	1.4	2.34	0.5	1.92	1.1	2.37	0.6
**3**-SnChl	1.88	1.3	2.40	0.4	1.94	1.2	2.25	0.4

**Table 7:** IC_50_ and PI values against MCF-7 cancer cells for **1**-**3**-Chl and **1**-**3**-SnChl and a series of tetraarylchlorins and Sn(IV) tetraarylchlorin complexes reported previously [15,16,17].

	Dark ^a^	Light ^b^	PI ^c^	Thorlabs LED ^b^	Time (Min) ^b^	Dose ^b^ (J·cm^−2^)	Ref.
Tetraphenylchlorin	>25	15.8	>1.6	M660L4	15	252	[15]
Tetrathien-2-ylchlorin	>25	3.5	>7.1	M660L4	15	252	[15]
Tetra-5-bromothien-2-ylchlorin	>25	2.7	>9.3	M660L4	15	252	[15]
Tetramethylthiophenylchlorin	>50	7.8	>6.4	M660L4	30	504	[17]
**1**-Chl	>25	12.3	>2.0	M660L4	20	336	--
**2**-Chl	>25	9.4	>2.7	M660L4	20	336	--
**3**-Chl	>25	6.1	>4.1	M660L4	20	336	--
Sn(IV) tetrathien-2-ylchlorin	>25	0.9	>27.8	M660L4	30	504	[16]
Sn(IV) tetramethylthiophenylchlorin	>50	3.9	>12.8	M660L4	30	504	[17]
**1**-SnChl	>25	1.5	>16.7	M660L4	20	336	--
**2**-SnChl	>25	1.1	>22.7	M660L4	20	336	--
**3**-SnChl	>25	1.0	>24.8	M660L4	20	336	--

^a^ Twenty-four-hour incubation dark cytotoxicity data and ^b^ phototoxicity data against MCF-7 cancer cell line upon exposure to a Thorlabs M660L4 (280 mW·cm^−2^, 20 min) LED. ^c^ PI = phototoxic index (dark and light IC_50_ values toxicity ratio).

**Table 8:** Log reduction values for **1**-**3**-Chl, **1**-**3**-SnChl and a series of tetraarylchlorins and Sn(IV) tetrachlorin complexes reported previously [15,16,17].

	Dye	Log_10_ Red. ^a^	Dye	Log_10_ Red. ^a^	LED	
	Conc. (µM)	*S. aureus*	Conc. (µM)	*E. coli*	Time (Min) ^b^	Ref.
Tetraphenylchlorin	2.5	1.18	15	0.02	60	[15]
Tetrathien-2-ylchlorin	2.5	7.22 @45 min	15	4.98	60	[15]
Tetra-5-bromothien-2-ylchlorin	2.5	7.42 @30 min	15	8.34 @45 min	60	[15]
Tetramethylthiophenylchlorin	2.5	10.6 @30 min	10	0.35	75	[17]
**1**-Chl	1	7.67 @45 min	5	2.20	75	--
**2**-Chl	1	7.67 @45 min	5	2.47	75	--
**3**-Chl	1	7.67 @45 min	5	2.65	75	--
Sn(IV) tetramethylthiophenylchlorin	2.5	10.5 @30 min	10	8.74 @60 min	75	[17]
**1**-SnChl	1	7.67 @15 min	5	3.90	75	--
**2**-SnChl	1	7.67 @10 min	5	4.24	75	--
**3**-SnChl	1	7.67 @10 min	5	4.36	75	--

^a^ Illumination time in minutes with a Thorlabs M660L4 (280 mW·cm^−2^) LED to achieve a Log_10_ reduction value corresponding to 0% cell viability is provided after the @ character. ^b^ Otherwise, the Log_10_ reduction value is for 60 or 75 min.

## Data Availability

Data sets are available from the corresponding author on request.

## Supplementary Materials

The following supporting information can be downloaded at: https://www.mdpi.com/article/10.3390/molecules28104030/s1, Figure S1: ^1^H NMR spectra of **1**-Chl, **3**-Chl and **1**-**3**-SnChl; Figure S2: MALDI-TOF MS data for **1**-Chl, **3**-Chl and **1**-**3**-SnChl; Table S1: Calculated full-width at half-maximum (FWHM) values for the B bands of **1**-**3**-Chl and **1**-**3**-SnChl in DMSO and 1% DMSO/H_2_O.

## Sample Availability

Samples of **1**-**3**-Chl and **1**-**3**-SnChl are available from the corresponding author.
