# Supplementary material for: The Photodynamic Anticancer and Antibacterial Activity Properties of a Series of meso-Tetraarylchlorin Dyes and Their Sn(IV) Complexes
Source: Molecules. 2023 May 11;28(10):4030. doi: 10.3390/molecules28104030 (PMC10222794; doi:10.3390/molecules28104030)
Supplement: Supplementary file 1 [file molecules-28-04030-s001.zip › molecules-2341382-supplementary.pdf]

molecules

MDPI

Supplementary Material

The Photodynamic Anticancer and Antibacterial Activity Properties of a Series of *meso*-Tetraarylchlorin Dyes and Their Sn(IV) Complexes

Rodah Soy [1], Balaji Babu [1,2], John Mack [1,*] and Tebello Nyokong [1]

[1] Institute for Nanotechnology Innovation, Rhodes University, Makhanda 6140, South Africa
[2] Department of Chemistry, SRM University-AP, Amaravati 522502, India
* Correspondence: j.mack@ru.ac.za; Tel.: +27-46-603-7234

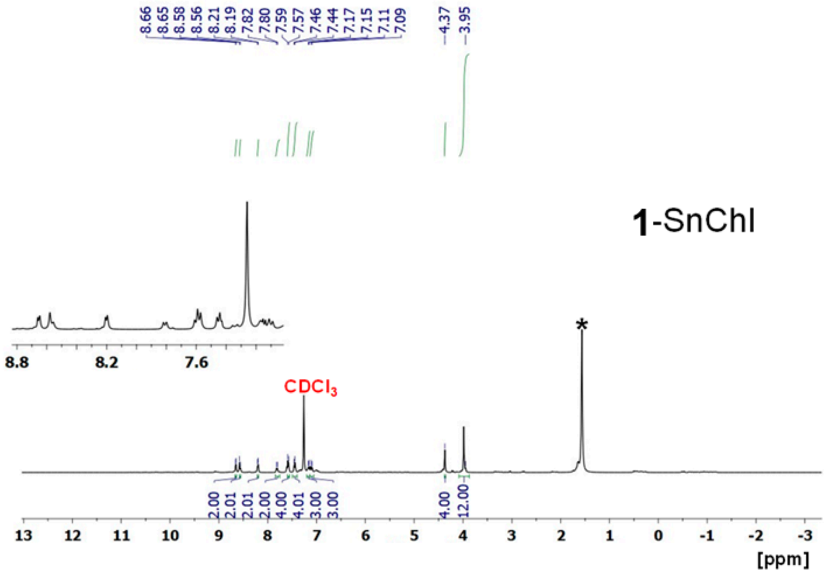

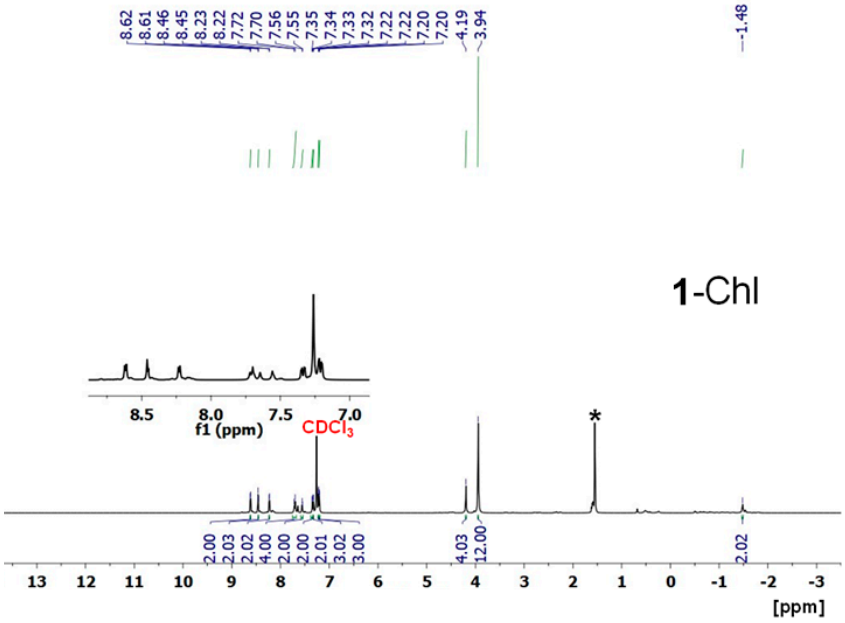

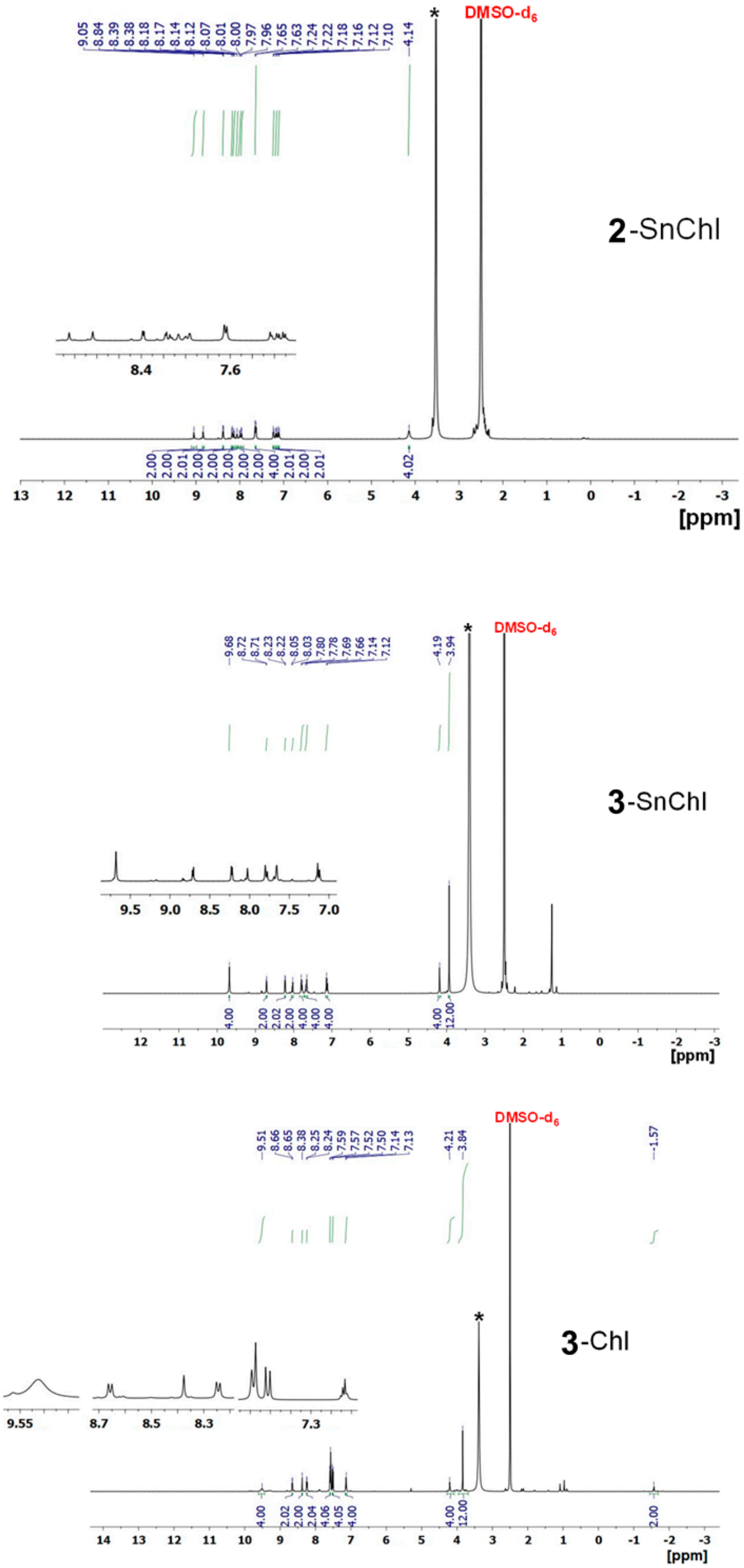

Figure S1. ^{1}H NMR spectra for **1**-Chl, **3**-Chl and **1**-**3**-SnChl. Black asterisks highlight the water peaks.

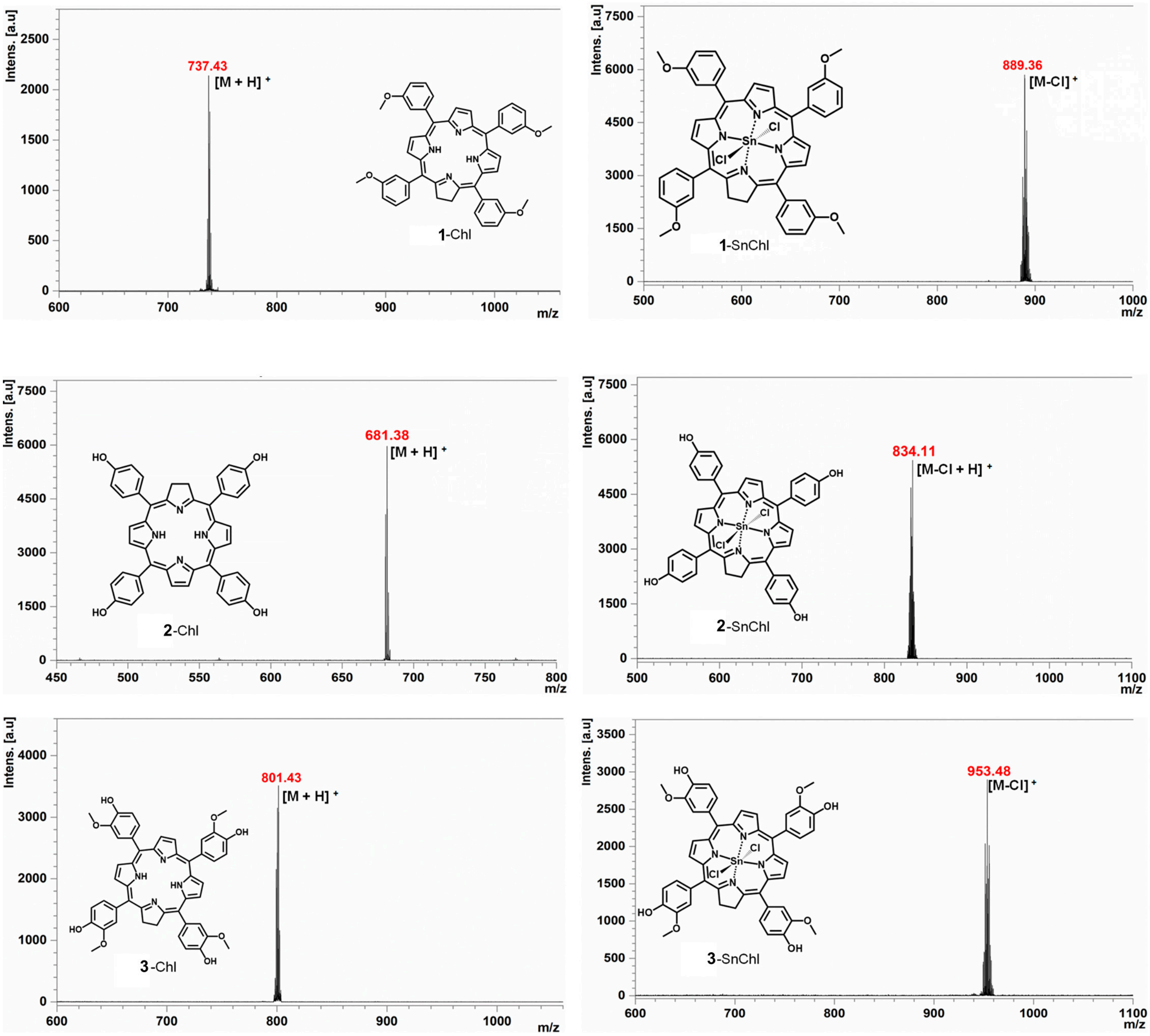

Figure S2: MALDI-TOF MS data for **1**-**3**-Chl and **1**-**3**-SnChl.

Table S1. Calculated full-width at half-maximum (FWHM) values for the B bands of **1**-**3**-Chl and **1**-**3**-SnChl in DMSO and 1% DMSO/H_2O.

	FWHM (nm) *	
	DMSO	1% DMSO/H_2O
**1**-Chl	20.7	33.2
**2**-Chl	28.1	43.2
**3**-Chl	24.8	41.4
**1**-SnChl	12.3	20.4
**2**-SnChl	29.1	32.4
**3**-SnChl	41.8	43.2
* calculated from fitting the B band with Origin software		
